# Protective Role of *Holothuria leucospilota* Extract Against Amyloid‐β Toxicity via Autophagy and Antioxidant Mechanisms in *Caenorhabditis elegans* Model

**DOI:** 10.1155/omcl/1471919

**Published:** 2026-10-13

**Authors:** Chanathip Kamonnat, Salinthip Thongdechsri, Pichnaree Kraokaew, Nakorn Niamnont, Prasert Sobhon, Krai Meemon

**Affiliations:** ^1^ Department of Anatomy Faculty of Science Mahidol University Bangkok 10400 Thailand mahidol.ac.th; ^2^ Department of Anatomy Faculty of Medical Science Naresuan University Phitsanulok 65000 Thailand nu.ac.th; ^3^ Department of Chemistry Faculty of Science King Mongkut’s University of Technology Thonburi Bang Mod Bangkok 10140 Thailand kmutt.ac.th

**Keywords:** Alzheimer’s disease, amyloid-β, autophagy, *Caenorhabditis elegans*, *Holothuria leucospilota*

## Abstract

Alzheimer’s disease (AD) is a major global health challenge characterized by cognitive decline and neuronal degeneration driven by amyloid‐β (Aβ) aggregation and oxidative stress. Given the limited efficacy and adverse effects of current pharmacological therapies, food‐derived bioactive compounds have gained increasing attention as potential preventive strategies against neurodegenerative disorders. In this study, we investigated the neuroprotective effects of *Holothuria leucospilota* extracts using a transgenic *Caenorhabditis elegans* model of AD. Our results demonstrated that *H. leucospilota* hexane extract (HLHE) at 500 µg/mL significantly delayed Aβ‐induced paralysis and improved chemotaxis behavior. Consistently, HLHE treatment reduced Aβ deposition and decreased both high‐ and low‐molecular weights of Aβ oligomers, as determined by automated Western blot analysis. Additionally, HLHE also reduced reactive oxygen species (ROS) levels. Transcriptomic and RT‐qPCR analyses revealed that HLHE enhanced autophagic activity through the upregulation of *bec-1* (autophagy initiation) and lysosomal genes (*cpr-1*, *cpr-2*, and *cpr-5*). Furthermore, HLHE activated antioxidant defense pathways via the insulin/IGF‐1 signaling (IIS) axis, specifically upregulating genes associated with SKN‐1 pathway (*pmk-1*, *skn-1*, *gst-4*, and *gst-10*) and HSF‐1 pathway (*hsf-1*, *hsp-16.1*, *hsp-12.6*, and *mtl-1*). HLHE also promoted nuclear localization of SKN‐1 and increased expression of its downstream target *gst-4*. Chemical analysis revealed that HLHE contained arachidonic acid (21.12%) and other bioactive lipid compounds as major constituents, suggesting a synergistic role in promoting autophagy and antioxidant responses. Collectively, this study suggests that HLHE confers neuroprotection against Aβ‐induced toxicity by activating autophagy and oxidative stress defense mechanisms, supporting its potential development as a food‐derived preventive or therapeutic agent for AD.

## 1. Introduction

Alzheimer’s disease (AD) is the global burden of disease associated with aging. AD is clinically characterized by cognitive dysfunction and degeneration of the neurons that are primarily caused by the extracellular aggregation of amyloid beta (Aβ_1–42_) peptide [1, 2]. Oligomeric Aβ_1–42_ peptide is widely recognized as the principle neurotoxic agent contributing to the pathogenesis of AD [3]. The Aβ_1–42_ oligomer is generated from amyloid precursor protein (APP) through sequential cleavage by β‐secretase and γ‐secretase, subsequently undergoing self‐assembly to form oligomers [4]. The aggregation of Aβ is linked to an imbalance between its synthesis and degradation processes [4, 5], leading to oxidative damage and neuronal dysfunction [6, 7].

AD progression is typically prolonged, accompanied by diverse comorbidities, and requires long‐term care [8]. In addition, the disease poses significant socioeconomic challenges, placing an increasing burden on the healthcare system and economies [9, 10]. Moreover, it is estimated that the number of dementia patients will rise from 57.4 million in 2019 to 152.8 million cases in 2050 [11]. Thus, AD needs attention as it becomes a major public health concern worldwide [12]. Reduction of Aβ production, aggregation, or accumulation in the brain is a promising anti‐AD strategy. Clearance of Aβ oligomers is crucial for protecting neurons and alleviating symptoms in AD patients [6]. Current clinical drugs do not directly target the removal of Aβ deposits, which are considered the primary cause of AD. Their therapeutic effects are largely palliative and vary among patients, while also being associated with potential side effects [13, 14].

Thus, there is an urgent need for research and the development of more effective anti‐AD medications. Natural crude extracts have attracted considerable attention as potential AD therapeutic candidates [15], owing to their low cytotoxicity and synergistic effects, whereby multiple constituents act on several cellular targets, enhance solubility, and improve pharmacokinetic properties [16]. Sea cucumbers have long held a prominent role in traditional food therapy across various Asian countries. Numerous studies have demonstrated that several sea cucumber species contain special compounds with various health benefits, functioning as antioxidants and anti‐inflammatory agents [17]. Correspondingly, our research group has previously explored its beneficial effects and found that crude extracts and purified compounds from the black sea cucumber, *Holothuria leucospilota*, attenuated dopaminergic neurodegeneration and reduced α‐synuclein aggregation in a *Caenorhabditis elegans* model of Parkinson’s disease [18, 19]. However, little is currently known about the efficacy of *H. leucospilota* extracts in AD treatment. In addition, *H. leucospilota* is abundant in tropical and subtropical waters, including the South China Sea and the broader Indo‐Pacific region (China, Thailand, Malaysia, Indonesia, Vietnam, and the Philippines). Unlike other commercially harvested species, *H. leucospilota* is not considered endangered [20, 21]. In contrast, the well‐studied species *H. scabra* has been reported to exert neuroprotective effects in a *C. elegans* AD model by reducing Aβ aggregation and oligomer formation, improving Aβ‐induced physiological impairments, extending lifespan, and reducing ROS levels [22]. However, the increasing demand for commercially valuable sea cucumber species has raised concerns regarding the depletion of their natural populations and the sustainability of their utilization [20, 21]. Therefore, investigating alternative and relatively abundant sea cucumber species with potential biological activities may provide valuable sources of bioactive compounds while reducing reliance on highly exploited species. In this context, *H. leucospilota* represents a promising but relatively understudied species for investigating potential neuroprotective effects against AD‐related pathology.

AD is a complex neurodegenerative disorder for which reliable in vitro models remain limited and often show low similarity to the pathological features observed in patients. Although in vivo models better replicate the complex biological characteristics of AD, particularly the mouse model [23], it is costly, time‐consuming, and associated with significant ethical concerns [24, 25]. In contrast, simple invertebrate models offer substantial experimental advantages for pharmacological screening. Among these, *C. elegans* is widely used due to its short lifespan, ease of maintenance, genetic tractability, and a high degree of genetic homology with humans (up to 83%) [26, 27]. Notably, multiple transgenic *C. elegans* strains expressing Aβ aggregation have been developed and can be used to assess differences in Aβ expression levels through both behavioral and molecular assays [28–30], making this model a powerful in vivo system for evaluating therapeutic compounds and investigating the mechanisms underlying Aβ‐induced neurotoxicity.

Based on the previous neuroprotective effects of *H. leucospilota* observed in a *C. elegans* model of Parkinson’s disease, we hypothesized that *H. leucospilota* extracts may also protect against Aβ‐induced toxicity in an AD model by modulating cellular defense mechanisms. Therefore, the present study aimed to investigate the neuroprotective effects of *H. leucospilota* extracts and their underlying mechanisms against Aβ toxicity in the *C. elegans* model of AD, suggesting their potential therapeutic application for the treatment of AD in the future.

## 2. Materials and Methods

### 2.1. Preparation of Crude Extracts From *H. leucospilota* and Chemical Composition Analyses of HLHE

The crude extracts of *H. leucospilota* were obtained following the method previously described by Sanguanphun et al. [31]. Briefly, the sea cucumber’s body walls were sliced into small pieces, preserved at −80°C, and subsequently lyophilized. The samples were extracted and fractionated using a sequential liquid–liquid extraction method with solvents of increasing polarity, including ethanol, n‐hexane, ethyl acetate, and n‐butanol. The stock solutions of crude extracts fraction from *H. leucospilota*, including ethanol extract (HLET), hexane extract (HLHE), butanol extract (HLBU), and aqueous extract (HLAQ), were dissolved in ddH_2_O. The experiments performed in *H. leucospilota* were ethically authorized by the Faculty of Science, Mahidol University–Institutional Animal Care and Use Committee (MUSC‐IACUC) following the protocol number MUSC66‐001‐631.

To discover the major components of HLHE (the most effective fraction), the gas chromatography–mass spectrometry (GC–MS) chromatogram analysis were assessed. The procedure of GC/MS analysis was conducted following our previous study [32]. Briefly, the HLHE extract was subjected to the Agilent 7890B/5977A series gas chromatography/mass selective detector (Agilent Technologies, Inc., Santa Clara, CA, USA) with a standard nonpolar column HP‐5 size of 30 mm × 0.25 mm ID × 0.25 μm film thickness. Afterward, the eluting chemical components were characterized by the National Institute of Standards and Technology (NIST) Mass Spectrometry Data Center.

### 2.2. Preparation of Transgenic *C. elegans* Model

All *C. elegans* strains obtained from the *Caenorhabditis* Genetics Center (CGC), University of Minnesota, USA, and maintained on nematode growth medium (NGM) plates fed with *E. coli* strain OP50 at 16°C, except the wild type strain (N2), which is maintained at 20 ^o^C. The transgenic *C. elegans* AD strains carrying human amyloid‐beta (Aβ_1–42_) used in this study were composed of CL2006 (*unc-54/*Aβ_1–42_), CL4176 (*smg-1*ts [*myo-3/*Aβ_1–42_ long 3^′^‐untranslated regions (UTR)]), and CL2355 (*snb-1/*Aβ_1–42_). In addition, wild‐type N2, CL802 (*smg-1* (cc546), and CL2122 (*mtl-2/*GFP) were used as controls for CL2006, CL4176, and CL2355, respectively. The transgenic GFP strains in this study include strains LD1 (ldIs7 [*skn-1*b/c::GFP + rol‐6(su1006)]) and CL2166 (dvIs19 [(pAF15)*gst-4*p::GFP::NLS] III). First stage synchronized larvae (L1) were used in experiments. To induce Aβ aggregation of CL2006 and nuclear localization of LD1 and CL2166 worms were maintained at 20°C throughout the experiment, while CL4176 and CL2355 worms needed an upshift to 25°C at L3 stage [33]. The experiments performed in *C. elegans* were ethically authorized following the protocol number MUSC66‐001‐631.

### 2.3. Lethality Assay

The toxicity of crude extracts from *H. leucospilota* were analyzed by investigating the lethality in N2 *C. elegans*. About 30–40 of L1 synchronized larvae were fed with *E. coli* OP50 containing 100, 250, 500, and 1000 µg/mL of HLET, HLHE, HLBU, and HLAQ extracts. For the control group, worms were fed with *E. coli* OP50 alone and *E. coli* OP50+ddH_2_O. The worms were incubated at 20°C and assessed for the survival rate of worms under stereomicroscopes after incubation for 24, 48, and 72 h.

### 2.4. Aβ‐Induced Paralysis Assay

Paralysis assay was applied to evaluate the impact of *H. leucospilota* extracts on Aβ aggregation in muscle cells of the transgenic *C. elegans* AD model (CL4176), which expresses the rollers phenotype, along with its control strain CL802. The paralysis assay was performed by modifying the method from our previous studies [22, 34, 35]. L1 larvae of CL4176 and CL802 were treated with HLET, HLHE, HLBU, and HLAQ extracts of *H. leucospilota* in doses of 100, 250, and 500 µg/mL mixed with *E. coli* OP50. For the control group, both CL4176 and CL802 were treated with *E. coli* OP50 alone and *E. coli* OP50+ddH_2_O. When the worms become L3 stage, they were upshifted to 25°C to induce Aβ aggregation. After 24 h, the number of paralyzed worms was counted every 2 h until 36 h. During observations, worms transitioning from a rolling phenotype to a state of immobility with a clear region around their heads were identified as paralyzed worms. The fraction and dose of *H. leucospilota* extracts that most effectively delayed paralysis were identified and selected for further experiments.

### 2.5. Chemotaxis Behavior Assay

Based on the results of the paralysis assay, HLHE extract at 500 µg/mL was the most effective fraction and dose. This extract was applied to evaluate the effect of *H. leucospilota* extract on the chemotaxis response of neuronal cells with Aβ toxicity in the transgenic *C. elegans* AD model. A chemotaxis assay was used to observe behavior alteration of the transgenic *C. elegans* CL2355 strain expressing human Aβ aggregation in neuronal cells. L1 synchronized CL2355 and CL2122 (control strain) were treated with HLHE (*E. coli* OP50+HLHE at 500 µg/mL) or without HLHE (*E. coli* OP50+ddH_2_O). L3 of CL2355 and CL2122 (control strain) were upshifted to 25°C to induce Aβ aggregation. After 36 h, the worms were collected and placed on 90 mm assay plates containing 1.9 % agar, 1 mM CaCl_2_, 1 mM MgSO_4_, and 25 mM phosphate buffer, pH 6.0 [34]. The assay plate was divided into two test and two control areas on the opposite side following the diagram in the Supporting Information 1: Figure S1. The test areas comprised an attractant solution (0.1% benzaldehyde in 100% ethanol) and sodium azide, while the control areas contained 100% ethanol and sodium azide. Subsequently, the worms were cultured at 25°C for 1 h, and the chemotactic index (CI) (Supporting Information 1: Figure S1) was calculated by using the formula: the number of worms at attractant areas minus the number of worms at control areas divided by the total number of worms.

### 2.6. Aβ Staining Assay

An Aβ staining assay was employed to investigate the effect of HLHE at 500 µg/mL on Aβ aggregation in the *C. elegans* AD model strain CL2006. L1 of N2 and CL2006 were treated with or without HLHE at 500 µg/mL. The worms in experimental plates were cultured on NGM plate at 20°C for 48 h and then transferred to floxuridine (FUDR)‐containing plates for an additional 48 h to prevent hatching of the worms. After that, the worms were stained with 50 µL of 1 mM 1,4–bis (3‐carboxy‐hydroxy‐phenylethenyl)‐benzene (X‐34) in 10 mM Tris–HCl pH 8.0, and destained worms were mounted with 10 µL of 2 µM sodium azide [22, 35] on 2% agarose slides. Fluorescence images were captured using a fluorescence microscope (Nikon eclipse upright microscope Eclipse Ci, Tokyo, Japan). The quantity of Aβ deposits stained with X‐34 in the head region for each worm was scored, with N2 *C. elegans* used as a control. Fluorescence images were analyzed using Image‐J software (National Institute of Health, Bethesda, MD, USA). The results were represented as the number of Aβ deposition and the % area of Aβ deposition in the head region of worm [36].

### 2.7. Automated Western Blotting of Aβ Species Assay

To evaluate the Aβ oligomer levels, transgenic *C. elegans* CL2006 treated with or without HLHE was analyzed by using Jess Automated Western Blot (Protein Simple’s Jess). Synchronized L1 of CL2006 were fed with or without HLHE at 500 µg/mL. The worms were cultured on NGM plate at 20°C for 48 h, and then transferred to FUDR plates for an additional 48 h. Subsequently, the worms were collected, quickly frozen in liquid nitrogen, and lysed with the RIPA lysis buffer supplemented with 10 mM phenylmethylsulfonyl fluoride (PMSF) and protease inhibitor cocktail (1x, Sigma–Aldrich, St. Louis, MO, USA). After homogenization, the samples were centrifuged at 12,000 rpm at 4°C for 5 min. The protein concentration of the collected supernatant was quantified using a bicinchoninic acid (BCA) assay kit. Subsequently, they were subjected to immunoblotting by using Jess Automated Western Blot (Protein Simple’s Jess), a capillary‐based immunoassay and fully automated Western Blot analysis with 12‐230 kDa Separation (Bio‐Techne, Minneapolis, USA). For the immunoblotting assay, Jess Automated Western Blot was conducted following the manufacturer’s instructions. Briefly, the protein samples from *C. elegans* were diluted with 0.1x sample buffer before mixing with fluorescent 5x master mix to yield the final concentration at 3 µg/lane. Subsequently, the protein samples were denatured before loading to the pre‐filled Jess microplate (5 µL/lane). Other reagents were dispensed following the diagram in the Supporting Information 1: Figure S2. For primary antibody of probe 1 and probe 2, anti‐Aβ (rabbit antihuman Aβ_1–17_ monoclonal antibody clone 6E10, My BioSource, San Diego, CA, USA) and anti‐α‐tubulin (rabbit mAb, 11H10, Cell Signaling Technology, Danvers, MA, USA) were diluted with Antibody Diluent 2 in the dilutions of 1:100 and 1:50, respectively. The goat anti‐rabbit secondary HRP conjugated (Bio‐Techne, Minneapolis, MN, USA) was used as a secondary antibody for anti‐Aβ and anti‐α‐tubulin. Notably, the primary and secondary antibodies of probe 1 (anti‐Aβ) were removed before replacing them with probe 2 antibodies (anti‐α‐tubulin) by using The RePlex Reagent Mix. Then, a capillary cartridge and a microplate were inserted into the Jess instrument to begin the run, which was carried out using Compass software (Bio‐Techne, Minneapolis, MN, USA). After the run was completed, the mean densities of Aβ immunoreactive bands were analyzed using Compass software.

### 2.8. Intracellular ROS Assay

To measure the impact of HLHE extracts on oxidative stress levels in the transgenic *C. elegans* AD model CL4176, the intracellular reactive oxygen species (ROS) analysis was detected using H_2_DCF‐DA (2^′^,7^′^‐dichlorodihydrofluorescein diacetate) staining. Green fluorescence is released when H_2_DCF‐DA is transformed into DCF by intracellular ROS. The ROS assay was performed by modifying the method from previous studies of our research group [32]. Synchronized L1 of AD worm CL4176 and non‐AD worm CL802 were treated with or without HLHE at 500 μg/mL. The worms were incubated at 16°C for 34 h and then induced Aβ aggregation by upshifting the temperature into 25°C for 36 h. Subsequently, the worms were stained with 50 μM H_2_DCF‐DA in the dark at room temperature for 1 h. Afterwards, the stained worms were washed with M9 buffer and mounted on 2% agar pad slides before taking the fluorescence pictures under a fluorescence microscope (Nikon eclipse upright microscope Eclipse Ci, Tokyo, Japan). The fluorescence intensities of DCF were assessed and analyzed using ImageJ software (National Institute of Health, Bethesda, MD, USA).

### 2.9. Lifespan of Transgenic *C. elegans* AD Model

To investigate the effect of HLHE on the lifespan of the *C. elegans* AD model, lifespan was measured in triplicate using at least 100 worms per transgenic line. The lifespan assay was performed by modifying the method from our previous studies [22, 32]. L1 worms of CL2006 and N2 were treated with or without HLHE at 500 μg/mL and maintained at 20°C on NGM plates for 34 h to become early L4 stage. Subsequently, the worms were transferred to FUDR plates and incubated at 25°C for 24 h. The dead, live, and censored worms were observed and continued counting daily until the last worm died. Worms were scored as dead when they exhibited no movement, no pharyngeal pumping, and did not respond to touch using a platinum loop. The data were represented as percent survival curves, which were statistically analyzed for *p*‐value by using the log‐rank (Mantel‐Cox) test.

### 2.10. Transcriptomic Analysis

To investigate gene expression levels and potential mechanisms associated with AD, L1‐synchronized CL4176 worms were treated with or without 500 µg/mL of HLHE at 16°C for 34 h, followed by an upshift to 25°C for an additional 36 h to induce Aβ aggregation. After incubation, worms were washed thoroughly with DEPC‐treated water, rapidly frozen in liquid nitrogen. The samples were extracted in accordance with the manufacturer’s procedure using the RNeasy mini kit (Qiagen, Hilden, Germany), and conducted following our previous study [19]. The quantity and purity of total RNA in the samples were measured by NanoDrop and agarose gel electrophoresis before being subjected to RNA‐sequencing (RNA‐seq) analysis by the BGI Tech Solutions (Hong Kong) Co., Ltd. (Tai Po, Hong Kong, China). The analysis processes were performed following the manufacturer’s instructions. The RNA sample was sequenced on DNBSEQ platform. The reference for *C. elegans* genes were conducted by the National Center of Biotechnology Information (NCBI) database version GCF_000002985.6_WBcel235 [37]. Differentially expressed genes (DEGs), both upregulated and downregulated, were analyzed using Poisson distribution model by comparing CL4176 treated with 500 μg/mL of HLHE to the control groups, with a *Q*‐value ≤0.05 for significance. To deeper understand the promising biological processes and pathways, the Kyoto Encyclopedia Genes and Genomes (KEGG) enrichment were examined and performed by Phyper on hypergeometric test with *Q*‐value ≤0.05. All results were downloaded from Dr. Tom, which is an online platform of BGI company.

### 2.11. Reverse Transcription‐Quantitative PCR (RT‐qPCR) Assay

Following the transcriptomic analysis, the expression of promising genes and pathways associated with anti‐AD effects were validated, with a particular focus on autophagy genes and those involved in the IIS pathway, using RT‐qPCR analysis. The procedure of RT‐qPCR was conducted following our previous study [19]. Briefly, CL4176 worms treated and untreated with HLHE at 500 μg/mL were collected for RNA extraction as mentioned earlier (2.10). Subsequently, cDNA was synthesized from 1 µg RNA using 4 µL of iScriptTM Reverse Transcription Supermix (Bio‐Rad, Hercules, CA, USA) in a total reaction volume of 20 µL, adjusted with nuclease‐free water. The reaction was carried out using T100 Thermal Cycler (Bio‐Rad, Hercules, CA, USA), and the obtained cDNA samples were stored at −20°C until required. For quantitative RT‐qPCR, 1 µL of cDNA were mixed in 5 µL SsoFast EvaGreen Supermix (Bio‐Rad, Hercules, CA, USA), 3 µL of DEPC water, and 0.5 µL of both forward and reverse primers (10 µL total volume) of interested genes as designated in the Supporting Information 1: Table S1. The cycling conditions were as follows: 1 × (30 s 95°C), and 44 × (5 s 95°C, 30 s 60°C). Changes in the expression of the target genes (Cq values) were calculated using 2^−(∆∆CT)^. For each sample, the fold change in the target gene were normalized to housekeeping gene (*act-1*) and relative to the control expression. Approximately 1000 worms participated in each group, with experiment performed triplicates.

### 2.12. Nuclear Localization Assay

The LD‐1 strain, which carries green fluorescence protein (GFP) tags under the *skn-1* promotor was used to examine the nuclear localization of SKN‐1 transcription factor. The synchronized L1 larvae were divided into three groups: a control group treated with *E. coli* OP50+ddH_2_O, a group treated with HLHE at 500 μg/mL, and a positive control group treated with 600 mM resveratrol. Then they were incubated at 20°C for 20 h in NGM plate, and subsequently transferred to FUDR plates for an additional 48 h. After that, adult worms were collected and mounted on 2% agarose slides for fluorescence imaging under a fluorescence microscope (Nikon eclipse upright microscope Eclipse Ci, Tokyo, Japan). Subcellular localization of SKN‐1 was categorized as low, medium, or high nuclear expression [38, 39]. The percentage of nuclear localization was quantified using ImageJ software (National Institute of Health, Bethesda, MD, USA). Worms exhibiting strong large nuclear fluorescence dots were identified as high nuclear, those with some nuclear fluorescence dots were classified as medium, and worms showing faint and small fluorescence dots were classified as low nuclear expression.

### 2.13. Fluorescence Intensity of *gst-4p*::GFP Gene Expression

The CL2166 strain, carrying GFP tags under the *gst-4* promotor (a downstream target of SKN‐1), was investigated in this assay [31]. The synchronized L1 larvae were treated with or without HLHE at 500 μg/mL at 20°C for 36 h in NGM plate, and then transferred to FUDR plates for an additional 48 h. After that, adult worms were collected and mounted on 2% agarose slides for fluorescence imaging under a fluorescence microscope (Nikon eclipse upright microscope Eclipse Ci, Tokyo, Japan). The fluorescence intensities of *gst-4p*::GFP was quantified and analyzed by using ImageJ software (National Institute of Health, Bethesda, MD, USA).

### 2.14. Statistical Analysis

All experiments were performed in triplicate. All data were statistically analyzed using GraphPad Prism software (GraphPad Software, San Diego, CA, USA). Experiments 2.3–2.6 were analyzed by one way‐ANOVA as well as the Turkey–Karma test for multiple comparisons. Data from other experiments were analyzed by a paired *t*‐test, except for the lethality assay and nuclear localization assay, which were analyzed by using Kaplan–Meier survival curves and log‐rank tests and two‐way ANOVA (Dunnett’s multiple comparisons test), respectively. Statistically significant was considered as a *p*‐value ≤ 0.05.

## 3. Results

### 3.1. Toxicity of *H. leucospilota* Extracts *in C. elegans* Model

Toxicity effects of *H. leucospilota* were investigated in the *C. elegans* model using lethality assay. The results revealed that the survival rates of wild‐type *C. elegans* (N2) at 24, 48, and 72 h in all treated groups comprising HLET, HLHE, HLBU, and HLAQ fractions at doses of 100, 250, 500, and 1000 μg/mL were not significantly different from those of the control group (Figure 1). The control group consisted of N2 worms treated with *E. coli* OP50 alone or *E. coli* OP50+ddH_2_O. Furthermore, no signs of toxicity were observed in the worms, as evidenced by survival rates remaining above 95% after 24, 48, and 72 h. Therefore, it can be concluded that all four fractions of *H. leucospilota* extracts, at dose up to 1000 μg/mL, did not exert any harmful effects on *C. elegans*.

**Figure 1:**
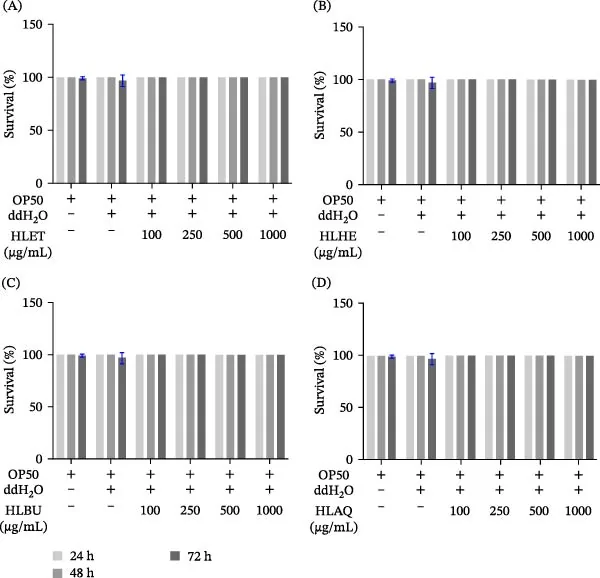
The toxicity of *H. leucospilota* extracts in wild‐type *C. elegans* (N2). (A–D) The percent survival of worms after treated with 100, 250, 500, and 1000 µg/mL of (A) HLET, (B) HLHE, (C) HLBU, and (D) HLAQ for 24, 48, and 72 h. The bar graph was displayed as mean ± SEM (three replicates, *n* = 30–40, number of worms per replicate). All data were compared with the control OP50+ddH_2_O group.

### 3.2. Effect of *H. leucospilota* Extracts on Paralysis Behavior of *C. elegans* Model

The paralysis assay was performed to assess the effects of *H. leucospilota* extracts on Aβ aggregation in the muscle cells of the transgenic *C. elegans* AD model (CL4176). The percentages of nonparalyzed worms in groups treated with 100, 250, and 500 µg/mL of HLET, HLHE, HLBU, and HLAQ fractions were compared with those of the control groups (Figure 2A,C,E,G). For the control group, AD worms (CL4176) and non‐AD worms (CL802) were treated with *E. coli* OP50 alone or *E. coli* OP50+ddH_2_O. The result showed that none of the non‐AD worm (CL802) exhibited paralysis, whereas the AD *C. elegans* (CL4176) treated with *E. coli* OP50 alone or *E. coli* OP50+ddH_2_O displayed paralysis within 24–36 h after Aβ induction. Notably, only the line graph representing the nonparalysis rate of worms treated with HLHE at 500 µg/mL (Figure 2C) was clearly separated from the control group (*E. coli* OP50+ddH_2_O), indicating a significant delay in paralysis in AD worms. The remaining fractions, on the other hand, did not exhibit any significant differences from their corresponding control groups.

**Figure 2:**
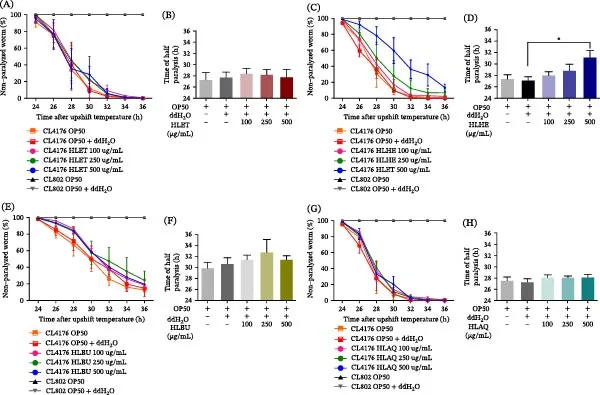
Effect of *H. leucospilota* extracts on paralysis behavior in muscular Aβ‐expressing *C. elegans* AD model (CL4176). The non‐Aβ expressing *C. elegans* (CL802) was used as a negative control. (A, C, E, and G) The percentage of nonparalyzed worms after exposure to 100, 250, and 500 µg/mL of crude extracts (A) HLET, (C) HLHE, (E) HLBU, and (G) HLAQ during 24–36 h after the upshifted temperature. The orange and red lines represented AD worm (CL4176) treated with OP50 alone and OP50 with ddH_2_O, respectively, whereas the pink, green, and blue lines represented treatment groups, including 100, 250, and 500 µg/mL of crude extracts, respectively. The control strain CL802 (non‐AD worm) treated with OP50 alone and OP50 with ddH_2_O was represented by the black and gray lines, respectively. (B, D, F, and H) The time of half‐paralysis rate (PT50) in CL4176 after treatment with 100, 250, and 500 µg/mL of (B) HLET, (D) HLHE, (F) HLBU, and (H) HLAQ. The data were presented as mean ± SEM (three replicates, *n* = 30–40, number of worms per replicate). All data were compared to CL4176 treated with OP50+ddH_2_O. The asterisk ( ^∗^) represented the significant difference between CL4176 treated with *H. leucospilota* crude extracts and CL4176 treated with control OP50+ddH_2_O,  ^∗^ *p*‐value < 0.05.

The time of half paralysis, represented by PT50 values (Figure 2B,D,F,H), was used to further evaluate the effects of *H. leucospilota* extracts. The results showed that the PT50 value of HLHE at 500 µg/mL (Figure 2D) was the highest, reaching 31 h after temperature upshift, and was significantly different (*p* < 0.05) from that of the control group (*E. coli* OP50+ddH_2_O group). This finding suggests that a longer time was required to induce paralysis in worms treated with HLHE. In contrast, no significant differences in PT50 values were observed between the control *E. coli* OP50+ddH_2_O group and any of the HLET, HLBU, and HLAQ treatment groups in all tested doses. Therefore, HLHE extract at 500 µg/mL was selected to perform in further experiments.

### 3.3. Effect of HLHE on Chemotaxis Behavior of *C. elegans* Model

The chemotaxis assay was performed to specifically observe the neuronal responses in the transgenic AD worms CL2355 strain expressing human Aβ aggregation in neuronal cells after treated with or without HLHE at 500 µg/mL. The result revealed that non‐AD worms (CL2122) treated with HLHE at 500 μg/mL showed no significant difference in the CI when compared to those treated with *E. coli* OP50+ddH_2_O (Figure 3). In contrast, AD worms (CL2355) treated with HLHE at 500 μg/mL exhibited a significantly higher CI value (*p* < 0.01) than the control group (CL2355 treated with *E. coli* OP50+ddH_2_O). These results suggest that HLHE at 500 μg/mL improved neuronal performance in CL2355 AD worms.

**Figure 3:**
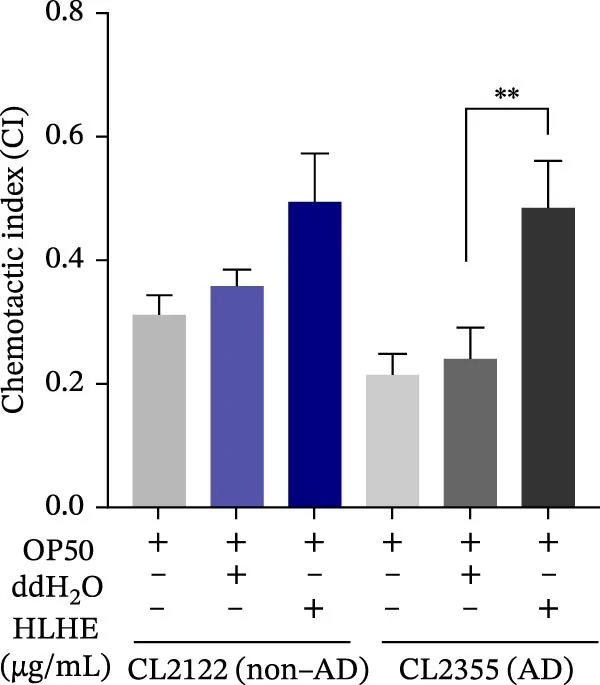
Effect of HLHE on chemotaxis response in *C. elegans* AD CL2355 model expressing Aβ in neuronal cells. The non‐AD *C. elegans* strain CL2122 was used as a negative control. The bar graphs represented the mean chemotactic index (CI) ± SEM in CL2122 and CL2355 *C. elegans* (three replicates, *n* = 50–150; number of worms per group per replicate). The asterisk ( ^∗^) represented the significant difference between 500 μg/mL of HLHE‐treated CL2355 and control OP50+ddH_2_O‐treated CL2355,  ^∗∗^ *p*‐value < 0.01.

### 3.4. Effect of HLHE on Aβ Deposition in *C. elegans* Model

In the Aβ staining (X‐34) assay, the impact of HLHE on Aβ deposition was examined in the AD model (CL2006), with N2 worms (wild type) serving as the control for baseline deposition. Aβ deposition was quantified in the head region at the level of the pharyngeal bulb. The results of fluorescent images revealed that wild‐type *C. elegans* (N2) treated with ddH_2_O did not exhibit Aβ deposition, while AD *C. elegans* (CL2006) treated with *E. coli* OP50 alone or *E. coli* OP50+ddH_2_O exhibited Aβ deposition (Figure 4A). The mean number of Aβ deposition in CL2006 AD worms treated with HLHE at 500 µg/mL was significantly decreased (*p* < 0.001), with a mean number of 20.62, compared to 44.12 in CL2006 treated with *E. coli* OP50+ddH_2_O (Figure 4B). Furthermore, the % area of Aβ deposition/head area in CL2006 treated with HLHE at 500 µg/mL was significantly reduced compared to CL2006 treated with *E. coli* OP50+ddH_2_O (*p* < 0.01; Figure 4C). These findings suggest that HLHE has the ability to inhibit Aβ deposition, leading to a reduction in toxic Aβ oligomer formation.

**Figure 4:**
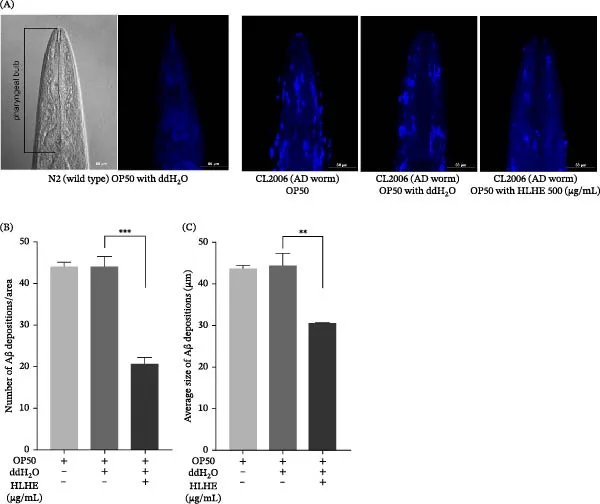
Effect of HLHE on Aβ deposition in transgenic *C. elegans* AD model. (A) Images of X‐34 staining, including bright field and fluorescent wild‐type N2 fed with OP50+ddH_2_O, AD worm CL2006 fed with OP50, AD worm CL2006 fed with OP50+ddH_2_O (control), and AD worm CL2006 fed with HLHE at 500 µg/mL. (B) Mean number of Aβ deposits in the AD CL2006 fed with HLHE at 500 µg/mL compared with control (OP50+ddH_2_O). (C) Mean size of Aβ deposits in the AD CL2006 fed with HLHE at 500 µg/mL compared with control (OP50+ddH_2_O). The data were presented as mean ± SEM (three replicates, *n* = 20, number of worms per replicate). The asterisk ( ^∗^) represented the significant difference between CL2006 treated with HLHE at 500 µg/mL and CL2006 treated with control OP50+ddH_2_O,  ^∗∗^ *p*‐value < 0.01 and  ^∗∗∗^ *p*‐value < 0.001.

### 3.5. Effect of HLHE on Aβ Levels in Transgenic *C. elegans* AD Model

The effect of HLHE on Aβ oligomerization was examined in the transgenic *C. elegans* CL2006 AD model using Jess Automated Western Blot and Compass software. The protein levels of Aβ oligomerization were analyzed automatically by Compass software and represented as the mean band densities of several sizes of Aβ proteins and *α*‐tubulin (Figure 5). The results revealed that the high molecular weight forms of Aβ oligomers approximately at 56 kDa (Aβ‐dodecamer) and 164 kDa (Aβ‐annuli), as well as low molecular weights at 13.5 kDa (Aβ‐trimer), 23 kDa (Aβ‐pentamer), and 48 kDa (Aβ globumers) of the AD worms treated with HLHE at 500 µg/mL were significantly decreased when compared to the control AD worms treated with *E. coli* OP50+ddH_2_O (56 kDa and 48 kDa: *p* < 0.01 and 13.5, 23, and 164 kDa: *p* < 0.05). These findings suggest that HLHE at 500 µg/mL may reduce the formation of toxic Aβ oligomers of both low and high molecular weights, likely by inhibiting their aggregation or promoting their degradation.

**Figure 5:**
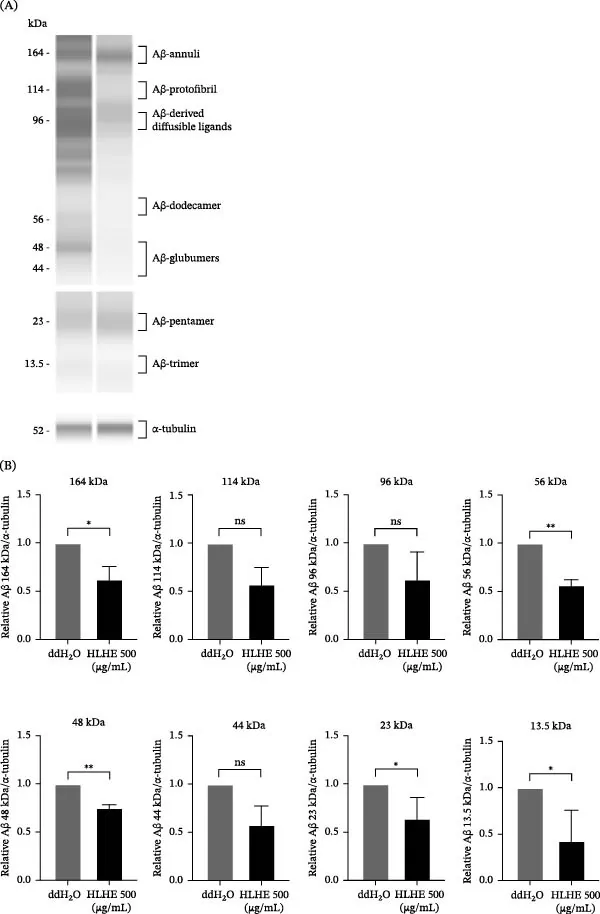
Effects of HLHE on Aβ oligomerization in transgenic *C. elegans* AD model detected by anti‐Aβ 6E10 and anti‐*α*‐tubulin antibodies. (A) Representative images of Jess automated western blot of Aβ species in the control ddH_2_O treated‐group and 500 µg/mL of HLHE‐treated CL2006 AD *C. elegans* were illustrated in the left and right panels, respectively. Quantification of relative Aβ/α‐tubulin band intensities were carried out with Compass software, The *α*‐tubulin at 52 kDa was used as a loading control. (B) The bar graphs represented the mean ± SEM of relative Aβ oligomers band intensities at 13.5, 23, 44, 48, 56, 96, 114, and 164 kDa normalized against *α*‐tubulin (three replicates, *n* = 1000–1500, number of worms per replicate). Asterisks ( ^∗^) indicated statistically significant differences between treated and untreated groups,  ^∗^ *p*‐value < 0.05 and  ^∗∗^ *p*‐value < 0.01, respectively.

### 3.6. Effect of HLHE on Oxidative Stress of *C. elegans* Model

The ROS assay was assessed to determine the intracellular ROS levels in the transgenic *C. elegans* AD model (CL4176) and control non‐AD worms (CL802). The results revealed that non‐AD worms treated with *E. coli* OP50+ddH_2_O showed low DCF fluorescence intensities, while CL4176 AD worms treated with *E. coli* OP50+ddH_2_O exhibited relatively high DCF fluorescence intensity to 134.4% (Figure 6A). In contrast, the DCF fluorescence of HLHE at 500 µg/mL was significantly decreased (*p* < 0.05) compared to the AD worms treated with *E. coli* OP50+ddH_2_O, with % relative DCF value of 110.3% (Figure 6B). Therefore, the results suggest that 500 µg/mL of HLHE may function as an antioxidant by dramatically decreasing the intracellular ROS levels in AD worms.

**Figure 6:**
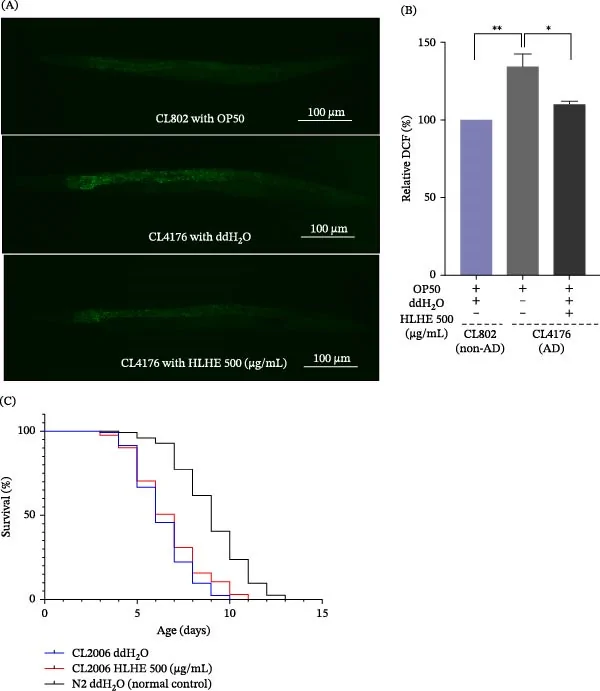
Effect of HLHE on oxidative stress and lifespan in transgenic *C. elegans* AD model. (A) The fluorescent image of intracellular ROS levels in CL4176 AD *C. elegans* and CL802 non‐AD *C. elegans* treated with or without HLHE at 500 μg/mL, the scale bar is 100 μm. (B) The percent relative DCF in CL802 and CL4176 *C. elegans* treated with or without HLHE at 500 μg/mL. The data were presented as mean ± SEM (three replicates, *n* = 30; number of worms per group per replicate). All data were compared with CL4176 worms treated with OP50+ddH_2_O. Asterisks ( ^∗^) indicated statistically significant differences between CL802 worms and HLHE‐treated CL4176 worms compared to control OP50+ddH_2_O‐treated CL4176 worms,  ^∗^ *p*‐value < 0.05 and  ^∗∗^ *p*‐value < 0.01, respectively. (C) The percent of survival curve was relevant to the lifespans of worms. For the CL2006 AD worms, the percent survival of control OP50+ddH_2_O‐treated groups and HLHE at 500 μg/mL were represented as blue and red lines, respectively. The data were performed in triplicates (*n* = 50; number of worms per group per replicate).

### 3.7. Effect of HLHE on the Lifespan of *C. elegans* Model

The lifespan of the *C. elegans* AD model (CL2006) treated with HLHE were evaluated using lifespan assay and compared with that of wild‐type (N2) worms as the normal phenotype control. The experimental groups, with or without HLHE treatment at 500 µg/mL, were assessed for survival rate and mean lifespan (Figure 6C and Table 1). The mean lifespan of untreated N2 worms was 8.954 ± 1.82 days, whereas that of the CL2006 AD worms without HLHE treatment (control) was 6.382 ± 1.508 days, indicating that AD worms had a significantly shorter lifespan than N2 worms (*p* < 0.0001). The mean lifespan of AD worms treated with 500 µg/mL HLHE was 6.697 ± 1.87 days, which was not significantly different compared to untreated AD worms. However, a slight upward trend was observed, suggesting a potential increase in lifespan.

**Table 1:** Mean lifespans, percentage of increased lifespans, and max. age of CL2006 AD *C. elegans* and wild‐type N_2_ *C. elegans* following treatment with HLHE at 500 μg/mL at 25°C.

Treatment groups	Mean lifespans ± SD (days)	% Increased lifespans	Max. age (days)	*p*‐Value
N_2_ + ddH_2_O	8.954 ± 1.82	—	12	*p* < 0.0001 ^∗∗∗∗^
CL2006 + ddH_2_O	6.382 ± 1.508	—	9	—
CL2006 + HLHE 500 μg/mL	6.697 ± 1.870	4.94	10	ns

*Note:* The asterisk ( ^∗^) indicates the significant differences of mean lifespans compared to CL2006 + ddH_2_O,  ^∗∗∗∗^ *p* < 0.0001.

Abbreviation: ns, not significant.

### 3.8. Effect of HLHE on DEGs Involved in Anti‐Alzheimer Activity

Transcriptomic analysis was performed to identify promising genes and pathways affected by HLHE treatment at 500 µg/mL in transgenic CL4176 AD worms, and the results were compared with those of the control group fed with *E. coli* OP50+ddH_2_O (Figure 7). The analysis revealed DEGs and KEGG pathway enrichment following HLHE treatment. Significant upregulation and downregulation of genes were observed in response to HLHE exposure (Figure 7A), with 1341 genes upregulated and 217 genes downregulated (Supporting Information 1: Figure S3A). The complete list of DEGs following HLHE treatment is provided in the Supporting Information 2. The major upregulated genes, including autophagy‐related genes and the genes associated with the insulin/insulin‐like growth factor signaling (IIS) pathway were selected for their potential roles in neuroprotection. HLHE treatment at 500 µg/mL significantly upregulated *cpr-1*, *cpr-2*, and *cpr-5*, key genes responsible for the autophagy pathway (Figure 7A). In addition, genes linked to SKN‐1 and HSF‐1, the key transcription factors associated with the IIS pathway, were prominently expressed after HLHE treatment (Figure 7A). Specifically, the upstream gene of the SKN‐1 oxidative stress response pathway (*pmk-1*) and the downstream genes of the HSF‐1 chaperone response (*hsp-12.6* and *mlt-1*) were significantly upregulated (Figure 7A). Furthermore, KEGG pathway enrichment analysis was performed to explore the promising pathways influenced by HLHE treatment (Figure 7B). The enriched pathways were primarily associated with neuroprotection, including lysosomal function, longevity regulation, xenobiotic metabolism, long‐term potentiation, dopaminergic synapse, and inflammatory mediator. These findings were consistent with KEGG pathway classification level 2 (Supporting Information 1: Figure S3B), which demonstrated potential pathways related to neurodegenerative diseases, nervous system, aging, and sensory system. Therefore, HLHE may modulate genes involved in lysosomal activity and SKN‐1‐ and HSF‐1‐mediated longevity pathways, contributing to enhance protein clearance, antioxidant defense, and heat shock responses that mitigate Aβ aggregation and toxicity in AD worms.

**Figure 7:**
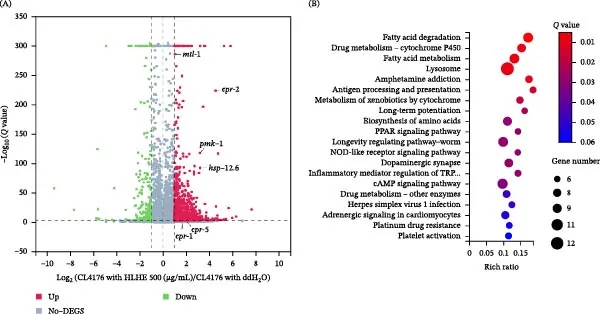
Effect of HLHE on differential genes expression in CL4176 AD worm. (A) The volcano plot of AD worms treated with HLHE at 500 μg/mL compared to ddH_2_O‐treated groups. The red dots indicated upregulated DEGs, while the green dots represented downregulated DEGs. The gray dots demonstrate non‐DEGs. (B) KEGG pathway enrichment of AD worms treated with HLHE at 500 μg/mL compared to ddH_2_O‐treated group. The sizes of bubbles refer to the number of genes. The colors of bubbles demonstrate the enriched significance (*Q* value), which range from red to blue, denoting high to low significance.

### 3.9. Effect of HLHE on the Expression of Genes Related to Anti‐Alzheimer Protection

To elucidate the underlying mechanisms of genes involved with the anti‐Alzheimer’s effects of HLHE, RT‐qPCR analysis was performed using the transgenic *C. elegans* CL4176 AD model. The expression of genes associated in the autophagy and IIS pathways was investigated in both HLHE‐treated and untreated AD worms, as listed in the Supporting Information 1: Table S1. The results demonstrated the significant upregulation of genes related to autophagy and the IIS pathway in AD worms treated with HLHE at 500 µg/mL, suggesting its potential neuroprotective effects. Specifically, the expression levels of autophagy‐related genes, including *bec-1* (1.16 ± 0.02 folds; *p* < 0.01), *cpr-1* (1.13 ± 0.02 folds; *p* < 0.01), *cpr-2* (1.43 ± 0.02 folds; *p* < 0.0001), and *cpr-5* (1.30 ± 0.01 folds; *p* < 0.0001) were significantly upregulated compared to the control group, indicating enhanced autophagic activity that may facilitate Aβ clearance (Figure 8A). Additionally, genes involved in the IIS pathway, including those associated with SKN‐1, DAF‐16, and HSF‐1 pathways, also exhibited transcriptional changes following HLHE treatment (Figure 8B). For the SKN‐1 pathway, *pmk-1* (1.19 ± 0.01 folds; *p* < 0.0001), *skn-1* (1.16 ± 0.03 folds; *p* < 0.01), *gst-4* (1.11 ± 0.03 folds; *p* < 0.05), and *gst-10* (1.17 ± 0.04 folds; *p* < 0.01) were significantly upregulated compared to the control group. In contrast, in the DAF‐16 pathway, only *mtl-1* mRNA showed significant upregulation (1.16 ± 0.03 fold; *p* < 0.01) after HLHE treatment, while *daf-16* and *sod*‐3 remained unchanged (Figure 8B). Notably, *mtl-1* functions as a co‐factor of both DAF‐16 and HSF‐1, implying potential cross‐talk between these pathways. Furthermore, the genes associated with the HSF‐1 pathway, including *hsf-1* (1.19 ± 0.01 folds; *p* < 0.001), *hsp-16.1* (1.25 ± 0.08 folds; *p* < 0.05), and *hsp-12.6* (1.30 ± 0.06 folds; *p* < 0.01), were significantly increased, indicating that HLHE at 500 µg/mL enhanced proteostasis and oxidative stress resistance in AD worms.

**Figure 8:**
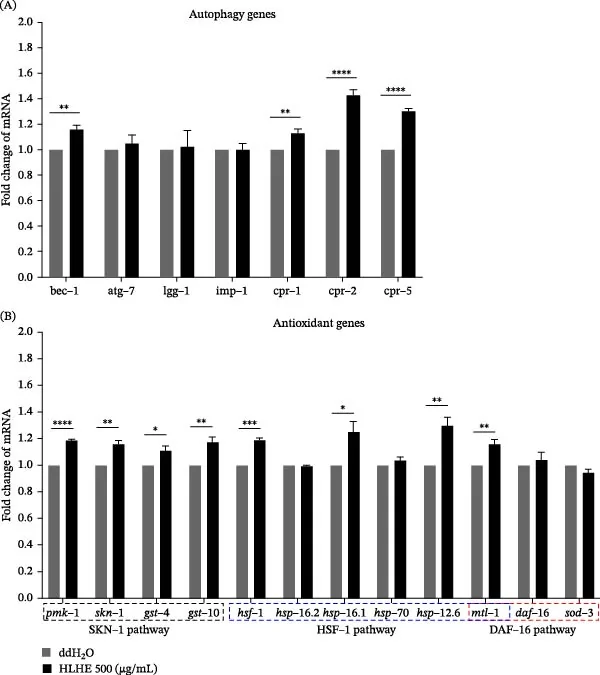
Effect of HLHE on transcriptional levels of genes associated with autophagy and IIS pathway in AD worm CL4176. (A) mRNA fold changes of the autophagy genes. (B) mRNA fold changes of IIS genes including SKN‐1, DAF‐16, and HSF‐1 pathways. The data were presented as mean ± SEM in triplicates. All data were compared with the control group (OP50+ddH_2_O). Asterisks ( ^∗^) indicated statistically significant differences between HLHE‐treated CL4176 worms and control OP50+ddH_2_O‐treated CL4176 worms,  ^∗^ *p*‐value < 0.05,  ^∗∗^ *p*‐value < 0.01,  ^∗∗∗^ *p*‐value < 0.001, and  ^∗∗∗∗^ *p*‐value < 0.0001, respectively.

Taken together, these results strongly suggest that HLHE provides anti‐Alzheimer effects by enhancing autophagy‐mediated clearance of Aβ aggregates in the *C. elegans* AD model. In addition, HLHE activated SKN‐1 and HSF‐1 signaling pathways, thereby promoting oxidative stress resistance and maintaining proteostasis in transgenic *C. elegans* AD model.

### 3.10. Effect of HLHE on SKN‐1 Nuclear Translocation

To further explore SKN‐1 activation, the transgenic worm strain LD1 was used as the model for investigating subcellular localization of SKN‐1 after treatment with or without 500 µg/mL HLHE. Worms treated with 600 mM resveratrol, a known SKN‐1 activator that promotes its nuclear translocation in intestinal cells, were used as a positive control [38, 39]. Worms treated with HLHE at 500 µg/mL showed a significant increase in SKN‐1 nuclear localization at 50.48% (*p* < 0.0001) when compared to the worms treated with *E. coli* OP50+ddH_2_O (Figure 9A,B). Similarly, worms treated with 600 mM resveratrol (positive control) exhibited a significant difference in SKN‐1 nuclear localization at 60% (*p* < 0.0001), when compared to the control group. Therefore, the results suggest that HLHE promotes SKN‐1 nuclear translocation in transgenic *C. elegans*, indicating its potential role in activating the SKN‐1 mediated oxidative stress response.

**Figure 9:**
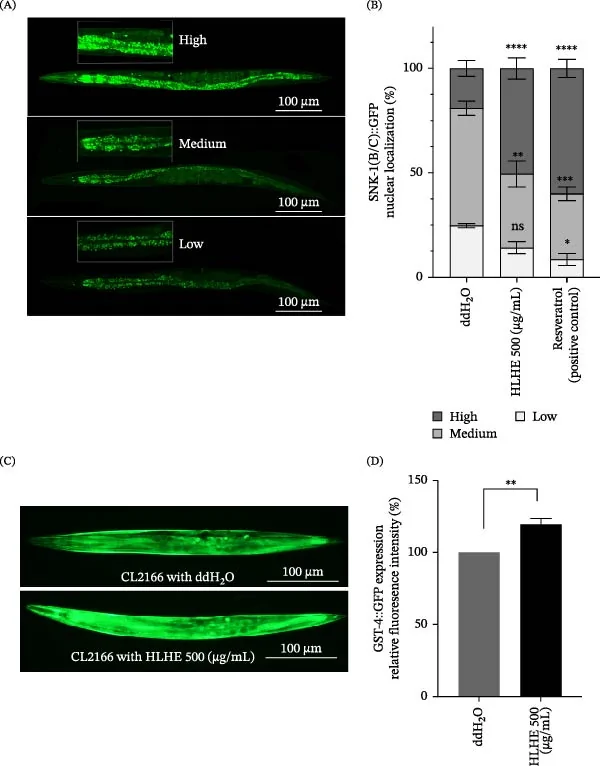
Effect of HLHE on elevated SKN‐1 nuclear translocation and *gst-4*p::GFP expression in *C. elegans*. (A) The fluorescent image of the worms LD1 (*skn-1*(b/c)::GFP) was categorized as high‐, medium‐, and low‐nuclear expressions of SKN‐1 with a scale bar of 100 µm. (B) The percentage of SKN‐1(B/C)::GFP nuclear localization of HLHE treatment, the data were presented as mean ± SEM in triplicates. (C) The fluorescent image of the worms CL2166 (*gst-4*p::GFP) showed the fluorescent intensity of the worms treated and untreated with HLHE at 500 µg/mL with a scale bar of 100 µm. (D) Quantification of relative fluorescence intensity (%) in CL2166 worms treated with HLHE compared to the untreated control, the data were presented as mean ± SEM in triplicates. All data were compared with the control group (OP50+ddH_2_O). Asterisks ( ^∗^) indicated statistically significant differences between HLHE‐treated worms and control OP50+ddH_2_O‐treated worms,  ^∗^ *p*‐value < 0.05,  ^∗∗^ *p*‐value < 0.01,  ^∗∗∗^ *p*‐value < 0.001, and  ^∗∗∗∗^ *p*‐value < 0.0001, respectively.

### 3.11. Effect of HLHE on *gst-4*::GFP Expression

The downstream effects of SKN‐1 activation were further examined by assessing *gst-4* expression in transgenic CL2166 (*gst-4*p::GFP) following HLHE treatment. Fluorescence imaging revealed that worms treated with 500 µg/mL HLHE exhibited higher *gst-4*p::GFP fluorescence intensity than the control worms treated with *E. coli* OP50+ddH_2_O (Figure 9C). Quantitative analysis showed that the relative fluorescence intensity in the HLHE‐treated group increased significantly to 119.5% (*p* < 0.01) when compared to the control group treated with *E. coli* OP50+ddH_2_O (Figure 9D). These results indicate that HLHE upregulated *gst-4* expression through the action of SKN‐1 in *C. elegans*, further supporting its role in enhancing oxidative stress resistance.

### 3.12. Chemical Composition of HLHE

The chemical constituents of HLHE were characterized using GC/MS chromatographic analysis (Table 2 and Supporting Information 1: Figure S4). The total ion chromatogram (TIC) of HLHE exhibited the relative abundance of chemical compounds across retention time (Supporting Information 1: Figure S4). The analysis identified 22 major compounds in HLHE, as listed in the Supporting Information 1: Table S2. Moreover, the details of the top 10 chemical constituents, including retention times (RT), molecular weights, chemical structures, peak areas, and percentage peak areas, were summarized in Table 2. Among these, arachidonic acid (21.12%) and cis‐10‐heptadecanoic acid (17.45%) were the predominant components, which correspond to a polyunsaturated omega‐6 fatty acid and a saturated fatty acid, respectively.

**Table 2:** The chemical components derived from HLHE by GC/MS analysis.

Compounds	RT (min)	Molecular weight	Chemical structure	Chemical compound	Peak area	% Peak area
1	40.300	304.24	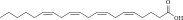	Arachidonic acid	2,055,076	21.12
2	40.643	268.24	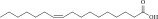	cis‐10‐heptadecenoic acid	1,698,258	17.45
3	43.953	390.317	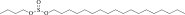	Sulfurous acid, butyl octadecyl ester	498,424	5.12
4	42.469	282.329	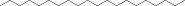	Eicosane	489,924	5.03
5	36.665	256.24	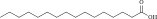	*n*‐hexadecanoic acid	486,709	5.00
6	40.354	302.225	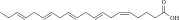	cis‐5,8,11,14,17‐eicosapentaenoic acid	481,525	4.95
7	38.915	284.272	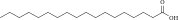	Octadecanoic acid	442,509	4.55
8	28.565	240.282	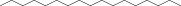	Heptadecane	376,443	3.87
9	38.722	282.256	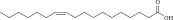	cis‐Vaccenic acid	346,765	3.56
10	41.265	346.291	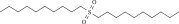	Di‐*n*‐decylsulfone	336,296	3.46

## 4. Discussion

Collectively, these findings provide compelling evidence that HLHE exerts anti‐Alzheimer’s effects by enhancing Aβ clearance and antioxidant defense, highlighting its potential as a natural preventive and therapeutic agent for AD. It significantly delayed Aβ‐induced paralysis, reduced Aβ toxicity, and improved neuronal behavior. HLHE also reduced Aβ deposits and toxic Aβ oligomers, likely through activation of the autophagy pathway and enhancement of antioxidant defenses via SKN‐1 and HSF‐1 transcription factors of IIS pathway. These findings suggest that HLHE mitigates Aβ pathology primarily by promoting protein clearance and reducing oxidative stress, highlighting its promising candidate for developing therapeutic treatments of AD. Moreover, GC–MS analysis identified arachidonic acid (21.12%) and cis‐10‐heptadecenoic acid (17.45%) as the predominant components of HLHE, which may contribute to its neuroprotective activity. A detailed comparison of the findings and experimental approaches between the present *H. leucospilota* study and the previous *H. scabra* study is provided in the Supporting Information 1: Table S3.

In this study, all fractions of *H. leucospilota* extracts exhibited no toxicity in *C. elegans*. Among them, the hexane fraction (HLHE) at 500 µg/mL demonstrated the strongest neuroprotective effect by significantly delaying Aβ‐induced paralysis in AD worms compared to other fractions. In addition, HLHE significantly reduced neuronal damage caused by Aβ aggregation in CL2355 worms, leading to improved neuronal performance as evidenced by enhanced chemotaxis behavior. These behavioral improvements align with previous findings where compounds capable of suppressing Aβ aggregation positively impacted neuronal behavior in *C. elegans* AD model. For example, the scorpion venom heat‐resistant peptide (SVHRP) inhibited Aβ oligomer formation and preserved chemotaxis behavior [40], while frondoside A from *C. frondosa* [34] and crude extracts of *H. scabra* [22] alleviated Aβ toxicity, improved chemotaxis, and delayed paralysis in CL2355 and CL4176 models. Given that *H. scabra* and *H. leucospilota* belong to the same genus, their similar neuroprotective potential is expected. Surprisingly, the CI of non‐AD worms also tended to increase following HLHE treatment, which may suggest its effect to enhance synaptic and cognitive function. Therefore, these results suggest that HLHE confers neuroprotection in *C. elegans* AD model by mitigating Aβ‐induced neuronal dysfunction, reducing toxic Aβ aggregation, and supporting neuronal health.

Abnormal behavioral improvement observed in HLHE‐treated worms could be attributed to the reduction of Aβ protein levels. This finding reinforces the notion that decreasing Aβ aggregation can protect against Aβ‐induced paralysis and improve chemotaxis behavior. Consistently, a significant reduction in both the number and size of Aβ deposits was observed in the muscle cells of treated AD worms. While previous studies primarily evaluated changes in the number of Aβ deposits, the present study additionally quantified the area of Aβ deposition, allowing to assess not only the number of deposits but also the extent of Aβ accumulation within the muscle cells. This aligns with previous studies demonstrating that extracts from *H. leucospilota* reduce *α*‐synuclein aggregation in *C. elegans* models of Parkinson’s disease [18]. Similarly, Kleawyothatis et al. [22] reported that *H. scabra* extracts significantly reduced Aβ deposits in the head region of CL2006 AD worms. Therefore, the combined assessment of Aβ deposit number and deposition area provides additional information on the effect of HLHE on Aβ accumulation.

Additionally, our analysis with the Jess Automated Western Blot revealed that HLHE treatment significantly reduced both high‐ (56 and 164 kDa) and low‐molecular weight (13.5, 23, and 48 kDa) Aβ oligomers, suggesting inhibition of Aβ aggregation and possible enhancement of oligomer degradation. These findings are consistent with those reported for *H. scabra* extracts in *C. elegans* AD models, which also reduced toxic Aβ oligomers at similar molecular weights [22]. Interestingly, HLHE treatment decreased the level of Aβ at 56 kDa—a dodecamer form considered as the most toxic oligomer capable of disrupting the neuronal membranes more strongly than the mature fibril. Previous studies suggest that Aβ dodecamers can form membrane pores, facilitating Ca^2+^ influx and triggering apoptotic signaling [41]. Importantly, the Jess Automated Western Blot was applied to characterize Aβ species across a broad molecular‐weight range and to examine the 30–60‐kDa Aβ species in greater detail. Detection of this species by conventional Western blotting may be challenging because its molecular weight is close to that of *β*‐actin or *α*‐tubulin, which can complicate accurate discrimination. In addition, our analysis identified Aβ species at ~44, 48, and 56 kDa that have not been previously characterized in the context of sea cucumber extract treatment in *C. elegans*. Taken together, these results suggest that HLHE, similar to *H. scabra* extracts, mitigates Aβ aggregation and oligomerization, while providing additional information on specific Aβ oligomeric species, particularly the potentially toxic dodecamer at 56 kDa. This further supports the neuroprotective potential of HLHE against Aβ toxicity.

Although both studies demonstrated anti‐Aβ effects, the present study provides more detailed characterization of Aβ oligomeric species and further investigated the underlying mechanisms associated with autophagy and antioxidant defense pathways. Such detailed characterization and mechanistic evidence are important for supporting and understanding the bioactive effects of sea cucumber extracts and their potential neuroprotective activity against Aβ toxicity.

The RNA‐seq analysis of *C. elegans* CL4176 AD model treated with 500 µg/mL of HLHE revealed significant modulation of gene expression, with 1341 genes upregulated and 217 downregulated. Notably, genes associated with autophagy and IIS pathway were prominently upregulated, suggesting the activation of neuroprotective mechanisms. The KEGG enrichment pathway further identified 12 lysosome‐related genes, including *cpr-1*, *cpr-2*, and *cpr-5*, indicating enhanced autophagic potential. To validate these transcriptional findings, RT‐qPCR was performed to examine autophagy‐related genes. The *bec-1* gene, homologous to mammalian Beclin 1, plays a pivotal role in autophagosome initiation by interacting with the lipid kinase Vps34 and other cofactors to promote the autophagic vesicle nucleation [42]. Furthermore, BEC‐1 serves as a crucial link between autophagy and apoptosis, with its inactivation leading to apoptotic cell death [43]. In addition, HLHE treatment significantly upregulated *cpr-1*, *cpr-2*, and *cpr-5*, encoding cysteine proteases analogous to cathepsin B. These proteases are integral to lysosomal function, facilitating the degradation of aggregated proteins and maintaining cellular homeostasis. Enhanced expression of these genes may bolster autophagic activity, promoting the clearance of toxic Aβ aggregates. This observation aligns with prior reports that upregulation of *cpr-2* and *cpr-5* preserves lysosomal integrity and ameliorates neurodegenerative markers during aging [34]. Therefore, these findings suggest that HLHE promotes autophagy pathway in transgenic *C. elegans*, resulting in reduced Aβ aggregation and improved neuronal behavior and paralysis in AD worms. Targeting Aβ aggregation through autophagy enhancement represents a promising therapeutic strategy, as it preserves the physiological roles of Aβ monomers, while promoting the clearance of neurotoxic aggregates [44].

Transcriptomic data also revealed upregulation of *pmk-1*, a gene encoding a p38 MAPK homolog, which is upstream of the SKN‐1 transcription factor. Activation of the *pmk-1* gene promotes SKN‐1 nuclear translocation and subsequently induces the expression of genes involved in oxidative stress responses and longevity. This activation suggests an enhanced defense mechanism against oxidative stress, a key protective mechanism against AD‐related neurotoxicity. Previous studies have demonstrated that the PMK‐1 pathway contributes to stress tolerance in *C. elegans*, highlighting its role in maintaining cellular integrity under stress conditions [45]. Consistent with the transcriptomic data, RT‐qPCR analysis confirmed significant upregulation of *pmk-1*, *skn-1*, and the downstream antioxidant genes, *gst-4* and *gst-10*, indicating robust activation of the SKN‐1 pathway. Additionally, HLHE treatment markedly increased the expression of *hsp-12.6* and *mlt-1*, downstream targets of the heat shock factor 1 (HSF‐1), suggesting an enhanced chaperone‐mediated proteostasis response. HSF‐1 activation induces heat shock proteins that assist in protein folding and protect against proteotoxic stress, relevant in mitigating Aβ‐induced toxicity. The HSF‐1 pathway has been implicated in lifespan extension by activating heat shock genes, thereby enhancing proteostasis and stress resistance [46]. However, no significant changes were observed in genes regulated by DAF‐16 pathway, implying that HLHE primarily exerts antioxidative and cytoprotective effects via HSF‐1 and SKN‐1 of IIS pathway. Supporting this, HLHE treatment increased SKN‐1 nuclear translocation and promoted *gst-4* expression in GFP‐reporter transgenic worms, confirming functional activation of this pathway. Taken together, these findings suggest that HLHE confers neuroprotection by activating SKN‐1 and HSF‐1 pathways, enhancing antioxidant defense and improving proteostasis. Through these mechanisms, HLHE likely reduces oxidative stress, mitigates Aβ aggregation, and alleviates neurotoxicity in the *C. elegans* AD model, offering valuable insights into multitargeted therapeutic strategies for AD.

GC–MS analysis revealed that HLHE contained 22 major chemical compounds (Supporting Information 1: Table S2), with arachidonic acid (21.12%) and cis‐10‐heptadecanoic acid (17.45%) identified as the predominant constituents. Cis‐10‐heptadecanoic acid has been reported to exhibit antitumor activity by inhibiting cell proliferation [47, 48]. Arachidonic acid, a polyunsaturated omega‐6 fatty acid, has demonstrated both neurotoxic and neuroprotective effects depending on its metabolic context. On one hand, arachidonic acid can be converted into prostaglandin E2 and D2, contributing to neuroinflammation; however, controlled neuroinflammation may also facilitate Aβ clearance [49]. Consistent with this dual role, our study found that HLHE promoted autophagic activity and reduced Aβ aggregation in the AD worms, suggesting that arachidonic acid may contribute to beneficial immune and clearance mechanisms. Moreover, free arachidonic acid serves as a retrograde messenger in synaptic signaling, enhancing the neurotransmitter release and supporting long‐term potentiation [49]. In addition to arachidonic acid, other HLHE components, such as *n*‐hexadecanoic acid (palmitic acid) and cis‐5,8,11,14,17‐eicosapentaenoic acid, may act as synergistically to balance oxidative and inflammatory responses [50, 51].

Although palmitic acid has been associated with cytotoxicity and oxidative stress at high concentrations [52], previous work from our group demonstrated that palmitic acid derived from *H. leucospilota* activated autophagy‐related genes (*bec-1* and *atg-7*) and detoxification genes (*gcs-1*, *gst-10*, and *gst-4*), supporting its protective role at appropriate doses [19]. Nevertheless, cis‐5,8,11,14,17‐eicosapentaenoic acid has been shown to enhance cognitive performance, reduce AD incidence, and counteract palmitic acid‐induced endothelial damage [53]. Collectively, these findings suggest that HLHE’s neuroprotective effects may arise from the synergistic interaction among multiple fatty acid components with complementary biological functions [16]. This concept aligns with previous studies emphasizing that natural product mixtures can act synergistically to target multiple AD hallmarks—reducing oxidative stress, decreasing Aβ deposition, and improving cognitive dysfunction.

## 5. Conclusion

This study demonstrated the neuroprotective effects of *H. leucospilota* hexane extracts (HLHEs) on alleviating Aβ‐induced Alzheimer’s pathology using transgenic *C. elegans* model. Treatment with 500 µg/mL HLHE significantly delayed paralysis and improved chemotaxis in AD worms, suggesting an amelioration of Aβ‐induced neurological impairments. HLHE also reduced Aβ deposition and oligomer accumulation in both high‐ and low‐molecular‐weight forms, accompanied by reduced ROS levels. Transcriptomic and RT‐qPCR analyses further revealed that HLHE activated autophagy‐ and antioxidant‐related pathways, particularly through upregulation of genes associated with autophagic activity and IIS pathway. GFP‐reporter assays confirmed the activation of SKN‐1 and HSF‐1, key regulators of oxidative stress resistance and proteostasis. Moreover, GC–MS analysis identified arachidonic acid and cis‐10‐Heptadecenoic acid as the predominant components of HLHE, which may contribute to its neuroprotective activity. These findings suggest that HLHE mitigates Aβ pathology primarily by promoting protein clearance and reducing oxidative stress, highlighting its promising candidate for developing therapeutic treatments of AD.

## Author Contributions

**Chanathip Kamonnat:** conceptualization, methodology, investigation, formal analysis, validation, data curation, visualization, writing – original draft. **Salinthip Thongdechsri:** methodology, investigation, validation. **Pichnaree Kraokaew:** validation. **Nakorn Niamnont:** methodology, validation. **Prasert Sobhon:** validation, writing – review and editing. **Krai Meemon:** conceptualization, validation, visualization, resources, supervision, funding acquisition, writing – review and editing.

## Funding

This study was funded by the National Research Council of Thailand (NRCT) and Mahidol University (Grant N42A660511) and partially supported by the Central Instrument Facility (CIF) (Grant AN‐KME‐3‐66‐02) of Faculty of Science, Mahidol University, Thailand.

## Ethics Statement

The experiments performed in *H. leucospilota* and *C. elegans* were ethically authorized by the Faculty of Science, Mahidol University‐ Institutional Animal Care and Use Committee (MUSC‐IACUC) following the protocol number MUSC66‐001‐631.

## Conflicts of Interest

The authors declare no conflicts of interest.

## Supporting Information

Additional supporting information can be found online in the Supporting Information section.

## Supporting information


**Supporting Information 1** Figure S1: Schematic illustration of chemotaxis assay. Figure S2: The diagram of the pre‐filled Jess microplate with 2 immunoassays. Figure S3: Impact of HLHE on the CL4176 AD worm’s differential gene expression. Figure S4: GC–MS chromatogram of the hexane fraction of *H. leucospilota* (HLHE). Table S1: The primers for RT‐qPCR analysis. Table S2: Chemical components derived from HLHE by GC/MS analysis. Table S3: Comparison of the experimental design, biological assays, and major findings between the previously published *H. scabra* study and the present *H. leucospilota* study.

**Supporting Information 2** Lists of upregulated and downregulated DEGs following HLHE treatment.

## Data Availability

The data that support the findings of this study are available from the corresponding author upon reasonable request.
