# Supplementary material for: Protective Role of Holothuria leucospilota Extract Against Amyloid‐β Toxicity via Autophagy and Antioxidant Mechanisms in Caenorhabditis elegans Model
Source: Oxid Med Cell Longev. 2026 Oct 13;2026:1471919. doi: 10.1155/omcl/1471919 (PMC13659408; doi:10.1155/omcl/1471919)
Supplement: Supplementary file 1 [file OMCL-2026-1471919-s001.docx]

**Supporting information for Manuscript**

**“Protective Role of *Holothuria leucospilota* Extract against Amyloid-β Toxicity via Autophagy and Antioxidant Mechanisms in *Caenorhabditis elegans* Model”**

**Figure S1.** Schematic illustration of chemotaxis assay. Chemotaxis assay was performed by using 0.1% benzaldehyde in 100% ethanol as an attractant and 100% ethanol as a control odorant. The worms were placed on the center of the assay plate and incubated at 25 °C for 1 h and the chemotaxis index (CI) was scored. CI value were defined as the number of worms at attractant points (T1+T2) - the number of worms at control points (C1+C2)/total number of worms.

**Figure S2.** The diagram of the pre-filled Jess microplate with 2 immunoassays. The probe 1 and probe 2 were designated as Aβ and α-tubulin proteins, respectively. The α-tubulin was used as a loading control.

**Figure S3.** Impact of HLHE on the CL4176 AD worms's differential gene expression. (A) The Number DEGS of AD worms treated with HLHE at 500 μg/mL compared to ddH_2_O-treated groups. The red bar indicated upregulated DEGs, while the blue bar represented downregulated DEGs. (B) KEGG pathway classification level 2 of AD worms treated with HLHE at 500 μg/mL compared to ddH_2_O-treated group.

**Figure S4.** GC-MS chromatogram of the hexane fraction of H. leucospilota (HLHE). (A) Chemical structure of arachidonic acid, the most abundant phytochemical compound identified. (B) Chemical structure of cis-10-heptadecanoic acid, the second most predominant constituent.

**Table S1.** The primers for RT-qPCR analysis

|  | Forward (5’ → 3’) | Reverse (3’ → 5’) |
| --- | --- | --- |
| **Autophagy mediators** | | |
| *atg-7* | TCTGCAGG TGGATGGTTCG | CTCGGCAAGGTCCATGTGTA |
| *bec-1* | AGATCTCAAAGCTGCGTGTG | AAAAGGCAGAATTCCAGCAGA |
| *lgg-1* | AATGGAAACCCAAAGCCCCT | AGGGGAGAAGAGCAACTTCG |
| *lmp-1* | CTCGCACCAACGAAGTTGTC | GTATCCGACGAGCACAACCA |
| *cpr-1* | CAATCGGCTGTTGAGACCCT | GGTGGCTTGGTCACGGATAA |
| *cpr-2* | ATCAAAGGGAGGACACGCTG | AAGAGTTGACAGCGAGCCAA |
| *cpr-5* | GACCGCTGGACATCAAGTCA | ACTTCCATGCCTGGATTGGG |
| **IIS pathway mediators** | | |
| *skn-1* | GTTCCCAACATCCAACTACG | TGGAGTCTGACCAGTGGATT |
| *pmk-1* | TCCGACTCCACGAGAAGGAT | CACGATATGTACGACGGGCA |
| *gst-4* | CCCATTTTACAAGTCGATGG | CTTCCTCTGCAGTTTTTCCA |
| *gst-10* | GTCTACCACGTTTTGGATGC | ACTTTGTCGGCCTTTCTCTT |
| *daf-16* | TTCCTGAAGAAGATGCTGACCTA | TATCGTCTGGCGATTCGGAC |
| *sod-3* | AGCACGCCTACTACTTGCAG | TGTACCGAGTGAAGTTCCCG |
| *mtl-1* | ATGGCTTGCAAGTGTGACTG | CACATTGTCTCCGCACTTG |
| *hsp-16.2* | GTCACTTTACCACTATTTCCGT | CAATCTCAGAAGACTCAGATGG |
| *hsf-1* | GGAGCCAATGAGCAAAAGCC | ATGAGGATCTGCGTTGGTGG |
| *hsp-70* | GCCGGTTGAAAAGGCACTTC | CGAGTTGATCCCCCAACCAA |
| *hsp-12.6* | AGACATGAATCTGTGCGCGA | TGGTTCCTTCGTCAGCCATC |
| *hsp-16.1* | CGTCCAGCTCAACGTTCTGT | TGGCTTGAACTGCGAGACAT |
| **Housekeeping gene** | | |
| *act-1* | ATCGTCACCACCAGCTTTCT | CACACCGGCAAATGAGTGAA |

**Table S2.** Chemical components derived from HLHE by GC/MS analysis.

| **Compounds** | **RT (min)** | **Molecular weight** | **Chemical structure** | **Chemical compound** | **Peak area** | **%peak area** |
| --- | --- | --- | --- | --- | --- | --- |
| 1 | 40.300 | 304.24 |  | Arachidonic acid | 2055076 | 21.12 |
| 2 | 40.643 | 268.24 |  | cis-10-Heptadecenoic acid | 1698258 | 17.45 |
| 3 | 43.953 | 390.317 |  | Sulfurous acid, butyl octadecyl ester | 498424 | 5.12 |
| 4 | 42.469 | 282.329 |  | Eicosane | 489924 | 5.03 |
| 5 | 36.665 | 256.24 |  | n-Hexadecanoic acid | 486709 | 5.00 |
| 6 | 40.354 | 302.225 |  | cis-5,8,11,14,17-Eicosapentaenoic acid | 481525 | 4.95 |
| 7 | 38.915 | 284.272 |  | Octadecanoic acid | 442509 | 4.55 |
| 8 | 28.565 | 240.282 |  | Heptadecane | 376443 | 3.87 |
| 9 | 38.722 | 282.256 |  | cis-Vaccenic acid | 346765 | 3.56 |
| 10 | 41.265 | 346.291 |  | Di-n-decylsulfone | 336296 | 3.46 |
| 11 | 34.270 | 254.297 |  | Octadecane | 331238 | 3.40 |
| 12 | 39.480 | 444.196 |  | Carbonic acid, octadecyl 2,2,2-trichloroethyl ester | 320974 | 3.30 |
| 13 | 43.413 | 322.199 |  | Butyl 2,5,8,11,14-pentaoxahexadecan-16-oate | 266508 | 2.74 |
| 14 | 37.160 | 334.254 |  | Sulfurous acid, butyl tetradecyl ester | 218697 | 2.25 |
| 15 | 40.523 | 298.095 |  | 4-Dehydroxy-N-(4,5-methylenedioxy-2-nitrobenzylidene)tyramine | 217192 | 2.23 |
| 16 | 45.824 | 352.407 |  | 2-methyltetracosane | 209136 | 2.15 |
| 17 | 48.174 | 222.126 |  | 1,2-Bis(trimethylsilyl)benzene | 189559 | 1.95 |
| 18 | 39.303 | 604.689 |  | Tritetracontane | 185494 | 1.91 |
| 19 | 40.704 | 207.083 |  | 2-Pyridinamine, N-(4,5-dihydro-5-methyl-2-thiazolyl)-3-methyl- | 156306 | 1.61 |
| 20 | 38.681 | 282.256 |  | 6-Octadecenoic acid, (Z)- | 145681 | 1.50 |
| 21 | 41.611 | 207.162 |  | N-(2-Acetylcyclopentylidene)cyclohexylamine | 141804 | 1.46 |
| 22 | 22.926 | 198.235 |  | Tetradecane | 137348 | 1.41 |

**Table S3.** Comparison of the experimental design, biological assays, and major findings between the previously published *H. scabra* study and the present *H. leucospilota* study.

| **Feature** | **Previous study: *H. scabra***  **(Kleawyothatis, et al., 2023)** | **Present study: *H. leucospilota*** |
| --- | --- | --- |
| **Sea cucumber species** | *Holothuria scabra* | *Holothuria leucospilota* |
| **Biological material** | Whole body (WB) and body wall (BW) | Body wall |
| **Main fraction investigated** | WBEA, BWEA, WBBU, BWBU, BWET | HLHE (hexane extract) |
| **Initial dose screening** | Mainly 500 µg/mL selected based on previous studies | 100, 250, and 500 µg/mL for paralysis screening. |
| **Selected effective dose** | 500 µg/mL | 500 µg/mL |
| **Toxicity assay** | NO | N2 worms tested at 100–1,000 µg/mL |
| **Aβ muscle model** | CL4176 and CL2006 | CL4176 and CL2006 |
| **Aβ neuronal model** | CL2355 | CL2355 |
| **Paralysis assay** | Yes | Yes |
| **Chemotaxis assay** | Yes | Yes |
| **Aβ deposition** | X-34 staining in CL2006 (investigated only Aβ number) | X-34 staining in CL2006 (investigated Aβ number and size) |
| **Aβ oligomer analysis** | Western blot | Jess automated Western blot |
| **ROS measurement** | Yes | Yes |
| **Lifespan assay** | CL2006, CL4176 and CL2355 | CL2006 |
| **Transcriptomic/RNA-seq analysis** | No | Yes |
| **KEGG pathway enrichment** | No | Yes |
| **RT-qPCR validation** | No | Yes |
| **SKN-1 nuclear translocation** | No | Yes, LD1 reporter strain |
| **gst-4::GFP reporter assay** | No | Yes, CL2166 |
| **Chemical analysis** | 1H-NMR | GC-MS |
| **Major compounds identified** | Triterpene glycosides/saponins and phenolic compounds discussed | Arachidonic acid and cis-10-heptadecanoic acid |
